# Periprosthetic Femoral Fractures and Their Surgical Outcomes Between 2011 and 2021: A Single-Centre Observational Study

**DOI:** 10.7759/cureus.28341

**Published:** 2022-08-24

**Authors:** Paul J Baggott, Mohamed Zubair Farook, Matthew Pritchard, Hardeep Singh, Anushruti Bista, Anshul Sobti, Ashwin Unnithan

**Affiliations:** 1 Trauma and Orthopaedics, Ashford and St Peter's Hospitals NHS Foundation Trust, Chertsey, GBR

**Keywords:** operative complications, complex trauma and reconstruction surgery, revision hip and knee surgery, periprosthetic fracture, femoral fracture

## Abstract

Introduction

Periprosthetic femoral fractures (PFFs) present a significant burden on the health service. The incidence continues to rise globally as a result of an ageing population and an increase in the number of primary hip and knee arthroplasties being performed. This is a 10-year, retrospective, observational study that aims to better understand the outcomes of PFF in our district general hospital.

Materials and methods

We identified the demographic information of patients who had a PFF and looked at how the American Society of Anesthesiologists (ASA) score, time to operation, length of stay, complications, and mortality data vary depending on where the fracture is sited and the operative management employed.

Results

During the period between January 2011 and March 2021, we identified 214 cases of PFF. The mean age was 82.5 years with a female preponderance of 76%. Between 2011-2016 and 2017-2021, the number of cases of PFF increased and patients with an ASA score of 3 or more increased from 43% to 73%. Length of stay was longer in the proximal PFF revision group than in the proximal PFF fixation group. Overall PFF mortality rates at 30 days, 90 days, and one year were 6%, 10%, and 15%, respectively.

Conclusion

Over the 10-year period, there was a significant increase in the incidence of patients presenting with PFF with multiple comorbidities. Mortality rates were lower in proximal PFF patients who underwent revision procedures rather than fixation. The patient demographics, complication rates, and mortality rates were comparable to similar studies across different countries.

## Introduction

Hip and knee arthroplasties have become increasingly popular procedures, largely due to excellent outcomes and ever-changing patient lifestyle demands [1]. As more of these procedures are undertaken, the number of periprosthetic femoral fractures (PFFs) is expected to rise [1-5]. In an ageing population with increasingly high rates of frailty and poor bone strength, these patients are at high risk of PFF even from relatively minor trauma [2].

PFF is a complex injury as both the joint prosthesis and fracture must be managed. Surgery is often faced with significant challenges and specialist skills are required [3]. PFF patients experience high rates of morbidity and mortality while healthcare resources are stretched by longer hospital stays and lengthy rehabilitation periods [4,5]. Currently, open reduction and internal fixation (ORIF) for stable implants or stem revision for loose femoral components represent the mainstay of management and this is guided by the Vancouver classification system [6]. Recently, the Universal Classification System (UCS) has been developed to incorporate the whole spectrum of periprosthetic fractures [7]. The purpose of this study was to identify the demographics of this group of patients at our district general hospital and analyse their surgical outcomes. We hope this data will add to the current literature on an emerging issue experienced by orthopaedic departments worldwide.

## Materials and methods

We retrospectively identified PFFs that had been managed surgically at a single centre between January 2011 and March 2021. Femoral fractures that occurred around a primary or revision total hip arthroplasty, hip hemiarthroplasty, and primary as well as revision total knee arthroplasty were included. Conservatively managed fractures were excluded from the study due to a lack of data. Electronic Patient Records (EPR) were reviewed for clinical data such as age, gender, laterality, length of stay (LOS), time until definitive surgery, American Society of Anesthesiologists (ASA) score, and comorbidities. Mode of internal fixation, revision arthroplasty and details of the prosthesis, fracture morphology, classification, nature of primary prosthesis, and complications were analysed from the hospital’s Picture Archive Communication System (PACS) system in addition to the clinical notes. Patient management was based broadly on the Vancouver and Universal Classification System, which incorporates the site of the fracture, stability of prosthesis and quality of surrounding bone. Patients were divided into four groups, according to management: hip fixation, hip revision, knee fixation or interprosthetic fixation. Outcome measures observed were medical and surgical complications, revisions and 30-day, 90-day and one-year mortality.

## Results

Patient demographics and fracture morphology

We identified 214 periprosthetic fractures in 209 patients during the study period. The mean age was 82.5 years (50-101) with a greater proportion of female patients (76%) and right-sided fractures (58%). The mean time until surgery was 2.2 days for fixation and 4.6 days for revision. The proportion of patients with an ASA score of 3 or more was 57%. The mechanism of injury was predominantly due to mechanical falls. A couple of cases were pathological fractures and one was high energy due to a fall from height. A total of 149 fractures were proximally sited, 45 distally and 15 were interprosthetic. Details of the patient demographics, time until surgery and length of stay are shown in Table 1. Of the 149 proximal fractures, 66 were around a cemented stem and 83 were around an uncemented stem. The type of implants used for the primary arthroplasty in the proximal PFF revision group and proximal PFF fixation group can be found in Table 2. As per the Vancouver Classification System or Duncan and Haddad’s Universal Classification System, of the 149 proximal fractures, 15% were B1, 48% B2, 7% B3 and 30% were C. Many patients had comorbidities including cardiovascular risk factors (57%), osteoporosis (10%) and multiple previous arthroplasty procedures (5%).

**Table 1:** Patient demographics (mean values) ASA: American Society of Anesthesiologists

	Patients (N)	Age (years)	Time until surgery (days)	Length of stay (days)	ASA of 3 or more (%)	Time since primary (years)
Proximal fixation	68	85	2.5	21.0	64	5.6
Proximal revision	81	81	4.6	21.3	56	6.8
Distal fixation	45	82	2.2	12.7	60	2.8
Interprosthetic fixation	15	82	1.7	13.1	40	3.1

**Table 2:** Type of primary arthroplasty in patients who have undergone proximal PFF revision and proximal PFF fixation PFF: periprosthetic femoral fracture

		Cemented	Uncemented	Hybrid	Reverse hybrid
Proximal hip revision	Total hip arthroplasty	10	37	10	1
	Hemi arthroplasty	4	14		
Proximal hip fixation	Total hip arthroplasty	16	29	12	2
	Hemi arthroplasty	1	7		

**Figure 1:**
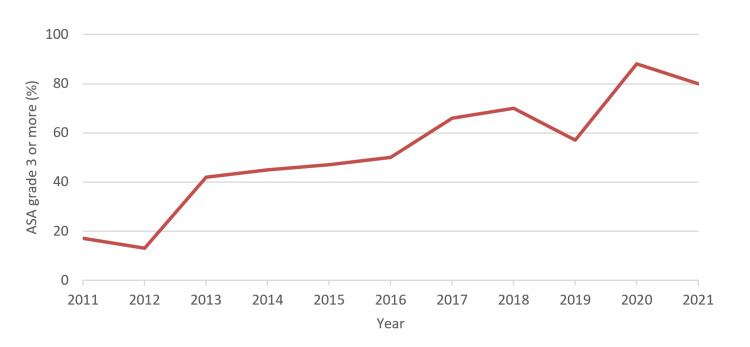
Proportion of patients with ASA grade greater than or equal to 3 ASA: American Society of Anesthesiologists

In our sample time period, we saw a rise in the number of cases per year. In the periods 2011 to 2016 and 2017 to 2021, the respective average cases per year were 5.3 and 7.3 for periprosthetic hip fixation, 5.0 and 10.8 for periprosthetic hip revision, and 3.1 and 5.4 for periprosthetic knee fixation. There was also a change in the ASA scores of patients over time, with the average proportion of the patients' ASA score of 3 or greater, increasing from 43% in the time period 2011-2016, to 73% in the period 2017-2021. The incidence of patients with an ASA grade of 3 or above is shown in Figure 1.

Management

Fixation was performed in 68 proximal fractures. Synthes locking compression plates (LCP) condylar plate (DePuy Synthes Companies, was most commonly used (45 out of 68). Eight patients were managed purely with cables. Eighty-one of the proximal fractures were managed with arthroplasty. Forty-four of these were managed with a Stryker Restoration Modular system. Of the 45 distal fractures, 35 underwent fixation using plates, predominantly with Synthes LCP condylar plate (LCP CP). Of the 15 interprosthetic fractures, 13 were managed with plates, most commonly with the Synthes LCP condylar plate.

The mean wait for surgery was 2.5 days for patients who underwent periprosthetic hip fracture fixation, 4.5 days for periprosthetic hip fracture revision, 2.1 days for patients with PFFs around a knee arthroplasty, and 1.7 days for interprosthetic fractures. The mean length of stay was 20.8 days, 19.8 days, 12.1 days and 13.1 days, respectively. Men were more likely to have a revision rather than fixation compared to women (31 out of 44 and 50 out of 105, respectively).

Failure, revision and complication rates

**Figure 2:**
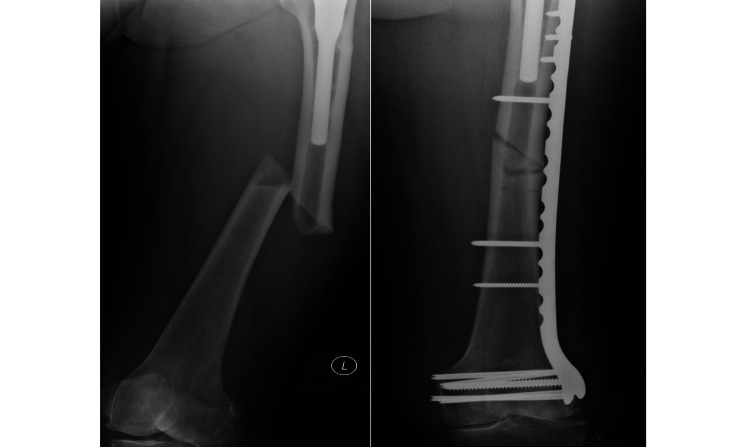
Before and after radiographs for a patient managed with plate fixation (which subsequently failed due to non-union)

**Figure 3:**
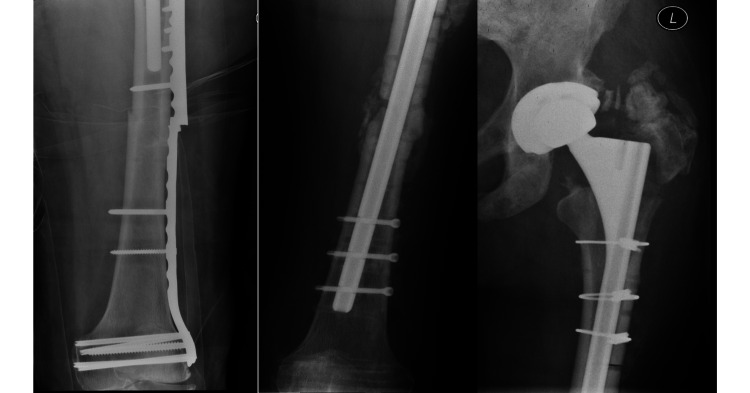
Radiographs for the same patient showing when the plate fixation failed due to non-union and was subsequently revised to a long interlocking femoral stem

In the proximal fixation group (N=68), four patients required further surgery because of hardware failure. They were managed using different fixation techniques or revision arthroplasty. One example was an 85-year-old female with a type C pathological fracture related to bisphosphonate use. She had a second plating operation after the first LCP failed due to non-union owing to too high a strain environment. She was subsequently revised to a long interlocking femoral stem. Radiographs are shown in Figure 2 and Figure 3.

In the proximal fracture group that underwent revision (N=81), six patients had dislocation. Two of the early dislocations (within one month of the procedure) were successfully managed with reduction under anaesthesia. Two others, one early and one at four months, required further revision of the acetabular component. One patient had a further PFF requiring revision with a Cannulok stem and cables. Two patients required return to the theatre due to infection, one for a further revision and one for a wound washout.

**Figure 4:**
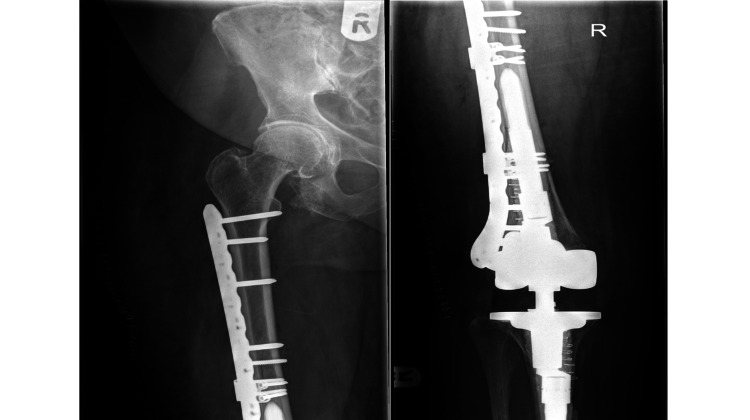
Radiographs for a patient managed with plate fixation (which subsequently failed due to non-union)

**Figure 5:**
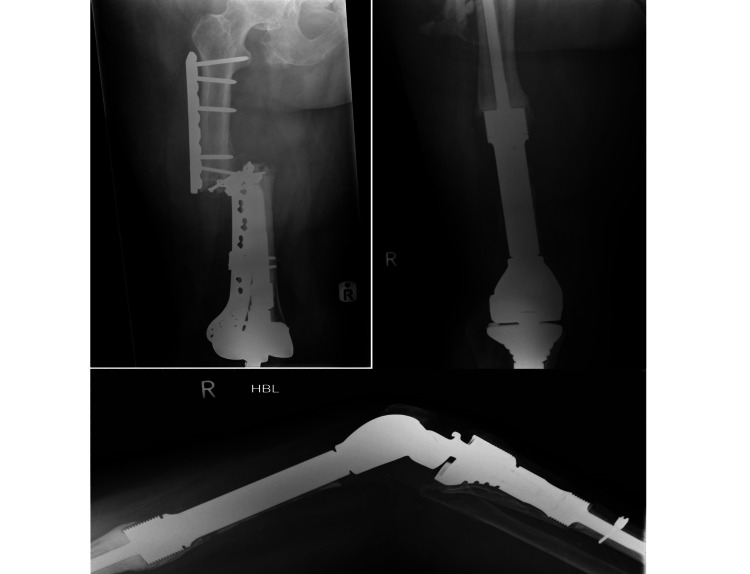
Radiographs for the same patient showing when the plate fixation failed due to non-union and was subsequently revised to a distal femoral replacement

Among the distal fixation group (N=45), three patients underwent revisions due to non-union. All three patients had periprosthetic femoral fractures around long revision femoral stems and had a fixation with plating. Two of them sustained a PFF less than a month following revision surgery and had a fixation with a 4.5 mm LCP and LCP CP, respectively. Both developed non-union due to too high a strain environment and fatigue fracture of the plate occurred around three months postoperatively. They were managed with distal femoral replacement. The radiographs for one example are shown in Figure 4 and Figure 5.

The interprosthetic group (N=15) did not require any further surgical intervention.

**Figure 6:**
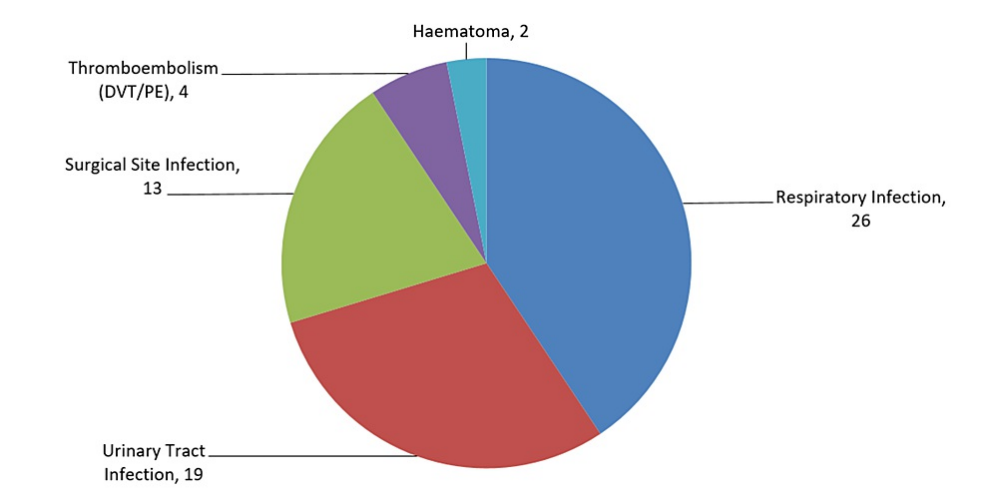
Complications following surgical management of PFF PFF: periprosthetic femoral fracture

The most common medical complication was respiratory infection (26 cases), followed by urinary tract infection (19 cases). The surgical and medical complications combined for all groups are shown in Figure 6.

Mortality Rates

The mortality rate for patients with any PFF was 6% at 30 days, 10% at 90 days and 15% at one year. There was one intraoperative death. The proximal femur fixation group experienced the highest 90-day mortality (16%) and one-year mortality (25%). The proximal femur revision group had the lowest 30-day mortality (3%), 90-day mortality (3%) and one-year mortality (7%). The distal femur fixation group had the highest 30-day mortality (10%). See all mortality data in Table 3.

**Table 3:** Mortality rates

	30-Day Mortality (%)	90-Day Mortality (%)	1-Year Mortality (%)
Proximal femur fixation	7	16	25
Proximal femur revision	3	3	7
Distal femur fixation	10	15	16
Interprosthetic fixation	7	13	13

## Discussion

The mean age of fracture in this study was 82.5 years old. Previous literature shows that old age is a risk factor for PFF, likely due to the increased prevalence of primary arthroplasty and poor bone strength [5]. We found that women were three times more likely to suffer from a PFF than men. Current evidence is inconclusive about a gender difference in PFF incidence. Abdel et al. and Finlayson et al. found in observational studies of proximal PFFs that there was no difference in risk due to gender. However, other studies agree that females have a greater preponderance, reporting between 60 and 80% of cases in females [3,8,9]. Women are known to have decreased bone strength compared to men, especially after menopause; this could explain their greater risk.

We found longer surgical waiting times in patients who underwent proximal PFF revision rather than proximal PFF fixation. We believe this may be because it took longer to find a surgeon with the required skills to perform revision surgery for a proximal PFF. A 2020 study found similar waiting times for proximal PFFs with any management, reporting a mean time of 3.7 days compared to 2.5 days and 4.6 days in our proximal PFF fixation group and revision group, respectively [10].

The mean length of stay in our study was 21 days for the proximal femoral and 12.7 days for the distal femur group. Other observational studies reported length of stay for any PFF as 17 days and 19 days, correlating increased length of stay with increased age of the patient [3,5].

Of the proximal PFF cases in our study, 83 were around an uncemented stem and 66 were around a cemented stem. The increased risk in uncemented stems has been reported as high as 10 times [1]. Foster et al. found similarly high rates of PFF in uncemented hip hemiarthroplasty stems (though found them to be used more frequently in older, frail patients) [11]. We concur with their recommendation to prefer cemented stems in older patients to reduce the risk of PFF.

The mortality rates across all PFFs at 30 days, 90 days and 1 year were 6%, 10% and 15%, respectively. These are in line with the one-year mortality rates in the current literature, reported as between 11% and 20% [5,12]. Moreover, the 30-day mortality is better than the 8.3% for native hip fractures published by the National Hip Fracture database [13]. Four previous studies found a rate at 30 days of 2.9% to 3.3%, lower than our study while one 2020 study found the rate to be higher at 6.4% in proximal PFF [3,5,10,12,14]. When subdivided, our 30-day mortality for the proximal PFF revision group was 3% but proximal PFF fixation and distal PFF fixation were 7% and 10%, respectively. Finlayson et al. reviewed the mortality rates in proximal PFF, finding 3.2% at 30 days, 5.8% at 90 days and 12.4% at one year. We found when divided between proximal PFF revision and fixation the one-year mortality rate was 7% and 25%, respectively. Much of the published literature does not differentiate the type of surgical management when analysing mortality rates. This is an advantage of our study which acknowledges a lower mortality rate in the proximal PFF revision group at 30 days, 90 days and one year when compared to the proximal PFF fixation group. We hypothesise that this is due to some bias toward fixation in the very frail patients with significant comorbidities and a B2 fracture.

Hoffman et al. reported in a cohort of proximal PFF patients that non-union rates were 5.9% and hardware failure occurred in 2% [15]. Other studies show non-union rates of between 3% and 10% [4,9]. Our non-union rate was 3.9% and our hardware failure rate was similar to Hoffmann et al. at 2.9%. The dislocation rate and deep infection rates in the revision subgroup were 6.5% and 3.2%, respectively, which also reflects the prevalence in the published literature [1,5].

An advantage of this study is that we can compare PFF data between the period 2011-2016 and 2017-2021. Our data recognise the growing incidence of PFF that is seen in the literature. Additionally, we observed that the ASA score of patients being treated for PFF is increasing. This is important as a higher ASA score correlates with an increased risk of morbidity and mortality. The increase in ASA scores seen over time could be due to a greater sensitivity to scoring. Unfortunately, though, it is not possible to reliably determine how significant the effect of this is.

This study could be improved by collecting and including data from other hospitals to provide a larger and more heterogeneous sample. Within this study, there were at least five surgeons across the time period who performed PFF fixation and revision surgery. Unfortunately, there is not enough data per surgeon to be able to reliably comment on the variation in procedure choice or outcomes. The study also does not include Patient Reported Outcome Measures (PROMs), which limits our ability to provide further detail to help with management choices. On the whole, patients who received fixation or revision for periprosthetic hip fractures were two different groups in terms of patient and fracture patterns. There were, however, some patients with B2 fracture patterns who could potentially have been treated with either revision or fixation. Unfortunately, with the retrospective data we have, it is not possible to draw definitive conclusions on the pros and cons of fixation versus revision.

## Conclusions

To our knowledge, this is the largest series of PFF reported from a district general hospital in England. A larger proportion of our patients were managed by fixation. Our rates of non-union, hardware failure following fixation and complications following revision arthroplasty were comparable to the literature. Our mortality rates were similar to other published studies and better than previous literature for hip fractures.

Our study agrees with previous literature that PFF is an increasingly prevalent issue impacting elderly patients with multiple comorbidities, and we found similar results to other comparable studies in terms of surgical outcomes.
